# Multidrug-resistant *Acinetobacter* Infection Mortality Rate and Length of Hospitalization

**DOI:** 10.3201/eid1301.060716

**Published:** 2007-01

**Authors:** Rebecca H. Sunenshine, Marc-Oliver Wright, Lisa L. Maragakis, Anthony D. Harris, Xiaoyan Song, Joan Hebden, Sara E. Cosgrove, Ashley Anderson, Jennifer Carnell, Daniel B. Jernigan, David G. Kleinbaum, Trish M. Perl, Harold C. Standiford, Arjun Srinivasan

**Affiliations:** * Centers for Disease Control and Prevention, Atlanta, Georgia, USA; † University of Maryland Medical School and Medical Center, Baltimore, Maryland, USA; ‡ Johns Hopkins University, Baltimore, Maryland, USA

**Keywords:** Acinetobacter infections, drug resistance, microbial epidemiology, infection control, research

## Abstract

*Acinetobacter* infections have increased and gained attention because of the organism’s prolonged environmental survival and propensity to develop antimicrobial drug resistance. The effect of multidrug-resistant (MDR) *Acinetobacter* infection on clinical outcomes has not been reported. A retrospective, matched cohort investigation was performed at 2 Baltimore hospitals to examine outcomes of patients with MDR *Acinetobacter* infection compared with patients with susceptible *Acinetobacter* infections and patients without *Acinetobacter* infections. Multivariable analysis controlling for severity of illness and underlying disease identified an independent association between patients with MDR *Acinetobacter* infection (n = 96) and increased hospital and intensive care unit length of stay compared with 91 patients with susceptible *Acinetobacter* infection (odds ratio [OR] 2.5, 95% confidence interval [CI] 1.2–5.2 and OR 2.1, 95% CI 1.0–4.3] respectively) and 89 uninfected patients (OR 2.5, 95% CI 1.2–5.4 and OR 4.2, 95% CI 1.5–11.6] respectively). Increased hospitalization associated with MDR *Acinetobacter* infection emphasizes the need for infection control strategies to prevent cross-transmission in healthcare settings.

*Acinetobacter* species are aerobic gram-negative bacilli that can cause healthcare-associated infections and can survive for prolonged periods in the environment and on the hands of healthcare workers (*1*–*3*). The proportion of healthcare-associated infections caused by *Acinetobacter* spp. has increased over the past decade in the United States (*4*). Furthermore, *Acinetobacter* infections have become increasingly difficult to treat because of the emergence of strains resistant to all drugs or all but 1 commonly prescribed antimicrobial drug (*5*–*7*). These multidrug-resistant (MDR) strains are sometimes susceptible only to polymyxins (colistin and polymyxin B), a class of antimicrobial drugs that has not been in widespread use for several decades and is more toxic than most currently used antimicrobial drugs. Outbreaks caused by MDR *Acinetobacter* have been reported in hospitals all over the world; more recently, they have become a serious problem in military medical facilities (*7*–*9*).

Although drug resistance of *Acinetobacter* is a recognized problem, the effect of MDR *Acinetobacter* infections on patient outcomes remains controversial. Previous studies on clinical outcomes of patients infected with *Acinetobacter* have yielded conflicting results and are limited by methodologic challenges that include small sample sizes, failure to control for severity of illness before infection, and failure to exclude patients colonized with *Acinetobacter* (*10*–*14*). Furthermore, most studies do not differentiate outcomes of patients infected with MDR *Acinetobacter* from those infected with drug-susceptible *Acinetobacter*. This finding leads to questions about the merits of targeting infection control measures to control MDR *Acinetobacter.* To determine the effect of MDR *Acinetobacter* infection on mortality rates, length of hospital stay, and length of intensive care unit (ICU) stay, the University of Maryland Medical Center, The Johns Hopkins Hospital, the Maryland Department of Health and Mental Hygiene, and the Centers for Disease Control and Prevention collaborated to perform a retrospective, matched, cohort study in Baltimore, Maryland. This is the first study that directly examines the effect of multidrug resistance on outcomes of *Acinetobacter* infections while controlling for severity of illness.

## Methods

### Study Design

A retrospective, matched cohort investigation was performed by using patient records from January 2003 through August 2004 from 2 tertiary care hospitals in Baltimore to examine outcomes of hospitalized patients with MDR *Acinetobacter* infection (exposure) compared with 2 unexposed reference groups: patients with susceptible *Acinetobacter* infection (susceptible references) and patients without *Acinetobacter* infection (uninfected references). We chose 2 reference groups to explore the effects of multidrug resistance and MDR *Acinetobacter* infection on patient outcomes (*15*). We defined MDR *Acinetobacter* as organisms resistant to all or all but 1 antimicrobial drug classes commonly prescribed to treat gram-negative infections. Susceptibility to polymyxins was not considered in these criteria because susceptibility testing for these drugs is not routinely performed. We defined susceptible *Acinetobacter* as organisms susceptible to >3 antimicrobial classes. Those patients infected with *Acinetobacter* that was susceptible to only 2 antimicrobial drug classes were excluded.

A computer-generated list was used to identify all persons from whom MDR *Acinetobacter* had been recovered from January 2003 through August 2004. Charts were then reviewed to determine, on the basis of criteria set forth by the National Nosocomial Infection Surveillance System (NNIS) (*16*), if the patient had 1 of the following infections caused by MDR *Acinetobacter*: bloodstream, pneumonia (respiratory tract), surgical site, urine, or sterile site other than blood. Admitted patients with both healthcare-acquired (i.e., infection was diagnosed >48 h after hospital admission) and community-acquired *Acinetobacter* infections (i.e., infection was diagnosed within 48 h of hospital admission) were included.

For the selection of matched susceptible references, microbiology records were reviewed to identify patients from whom susceptible *Acinetobacter* had been recovered from January 2003 through August 2004 at each institution. NNIS definitions were then applied to identify *Acinetobacter*-infected patients who were included in the study (*16*). To ensure that susceptible references and their matched MDR *Acinetobacter* patients had a similar exposure time, the susceptible references had to have a preinfection length of hospital stay within 5% of the matched MDR *Acinetobacter* patient’s preinfection length of stay. Patients infected with MDR *Acinetobacter* were matched to susceptible references from the same institution.

The second reference group, uninfected patients, included patients without *Acinetobacter* infection who had a length of hospital stay (time between admission and discharge) at least as long as the preinfection length of stay of the respective matched patient infected with MDR *Acinetobacter*. The matched uninfected patient also had to be present in the ward where the patient infected with MDR *Acinetobacter* was located within 30 days of becoming infected with MDR *Acinetobacter*.

Data abstracted from medical records included demographic information; presence of prior and concurrent medical conditions; dates of admission and discharge to the ICU and hospital; date and time of *Acinetobacter* culture; *Acinetobacter* antimicrobial susceptibility pattern; length of stay before infection (exposure time); presence or absence of concordant antimicrobial therapy on the day of the *Acinetobacter* culture (based on the susceptibility pattern of the organism); patient status on day of discharge; and date and cause of death if applicable. Data were collected on the following outcomes: in-hospital mortality rates and total number of days in hospital and ICU after the index day (i.e., length of stay after the exposure). We defined the index day as either the day of hospitalization on which *Acinetobacter* infection was diagnosed for patients infected with MDR *Acinetobacter* and for susceptible reference patients or the same day of hospitalization for uninfected patients. For example, if an MDR *Acinetobacter* infection was diagnosed on hospital day 15, that would be the index day for the MDR *Acinetobacter* patient and for the matched, uninfected patient.

To control for severity of illness before *Acinetobacter* infection, data were collected to calculate a modified Acute Physiology and Chronic Health Evaluation III (APACHE) score (*17*,*18*) ≈48 h before the index day. Our modified APACHE score did not include blood pH, pulmonary arterial oxygen saturation, pulmonary arterial gradient, urine output, or scoring for neurologic abnormalities. These parameters were excluded because they were not uniformly available for all patients in the study, particularly for those not in the ICU. To control for underlying disease, a Charlson comorbidity index (*19*) was calculated by using data from the medical history recorded on the chart.

### Statistical Analysis

All data were collected on standard forms, entered into an Access database (Microsoft, Redmond, WA, USA) and analyzed with SAS software (SAS Institute, Cary, NC, USA). Demographic data were analyzed with Mantel-Haenszel relative risks and 95% confidence intervals (CIs) to compare categorical variables and the Wilcoxon 2-sample test with t approximation to compare continuous variables. Matched univariate analysis was performed by using conditional logistic regression. Multivariable analysis controlling for severity of illness with the APACHE score and underlying diseases with the Charlson index was performed by using conditional logistic regression to evaluate in-hospital mortality rate and hospital and ICU length of stay.

To create a dichotomous variable for ICU and hospital lengths of stay, we compared the number in each comparison group that had a length of stay greater than the combined mean of the 2 groups being compared. For example, the combined group of MDR *Acinetobacter*–infected and susceptible *Acinetobacter* references had a mean hospital length of stay of 23 days after the index day (day of infection). The number of MDR *Acinetobacter* patients who had a hospital length of stay >23 days was then compared with the number of susceptible references who had a hospital length of stay >23 days, while controlling for severity of illness and associated underlying diseases in the multivariable model. We chose to compare against the mean rather than the median length of stay to account for outliers. Unmatched MDR *Acinetobacter*–infected and reference patients were excluded from the groups before the mean was calculated. A linear regression model and an ordinal logistic regression model were also attempted; however, these models could not be used because the outcome data were not normally distributed and could not be transformed appropriately for linear regression and because the assumptions for the ordinal logistic regression model could not be satisfied.

To examine the effect of discordant empiric antimicrobial drug therapy on clinical outcomes, multivariable analysis was performed on the MDR *Acinetobacter* patients alone; the exposure evaluated was concordant versus discordant empiric antimicrobial drug therapy. Outcomes included mortality rate, length of hospital stay, and ICU stay. We defined discordant empiric antimicrobial drug therapy as the administration of antimicrobial drug(s) to which the *Acinetobacter* strain was not susceptible. Concordant empiric antimicrobial drug therapy is defined as the administration of antimicrobial drug(s) to which the *Acinetobacter* strain was susceptible. APACHE and Charlson index scores were included in the model to control for their effect on outcomes.

Effect modification between MDR *Acinetobacter* reference groups and APACHE and Charlson index variables was evaluated by testing appropriate interaction terms for statistical significance. All statistical tests were 2-tailed; a p value <0.05 was considered statistically significant.

## Results

From January 2003 through August 2004, a total of 166 patients had cultures that grew MDR *Acinetobacter*, and 96 (58%) met the NNIS criteria for an MDR *Acinetobacter* infection (*16*). Of the MDR isolates, 88 (92%) were not susceptible to carbapenems.

### MDR *Acinetobacter*–infected Patients Compared with Infected References

We identified 91 reference patients infected with susceptible *Acinetobacter* who had similar lengths of hospital stay before the index day as MDR *Acinetobacter*–infected patients. Five MDR *Acinetobacter*–infected patients were excluded from the analysis because reference patients could not be identified due to a lack of susceptible references who were hospitalized for long periods. MDR *Acinetobacter* patients and susceptible references were similar in age and sex; however, MDR *Acinetobacter* patients had higher baseline mean APACHE and Charlson index scores than susceptible references (Table 1). The distribution of culture sites among MDR *Acinetobacter* and susceptible reference patients was similar; ≈50% of each group had respiratory infections, 31% in each group had bloodstream infections, and <10% of both groups had surgical wound, urinary tract, or other sterile site infections (p not significant for all comparisons). A total of 78 (81%) MDR *Acinetobacter* infections and 73 (80%) susceptible *Acinetobacter* infections were identified >48 h after hospital admission and thus met criteria for nosocomial infection.

**Table 1:** Comparison of baseline characteristics of patients with multidrug-resistant (MDR) *Acinetobacter* infection with those with susceptible *Acinetobacter* infection and those without *Acinetobacter* infection, Baltimore hospitals, 2003– 2004*

Characteristic	MDR *Acinetobacter,* n = 96	Susceptible *Acinetobacter*, n = 91	p values for MDR *Acinetobacter* vs. susceptible, n = 187	Uninfected, n = 89	p values for MDR *Acinetobacter* vs. uninfected, n = 185
Median age, y	54	52	0.61	52	0.97
Age range, y	14–83	12–85		17–90	
Sex, % male	67	57	0.78	65	0.96
Mean modified APACHE III score (median)	41 (42)	36 (33)	0.02	32 (28)	<0.001
Mean Charlson index (median)	3.9 (3.0)	2.8 (2.0)	0.01	2.5 (2.0)	<0.01

*APACHE III, Acute Physiology and Chronic Health Evaluation III.

Matched univariate analysis of patient outcomes showed that MDR *Acinetobacter*–infected patients had higher mean lengths of hospital stay and ICU stay after the index day than susceptible and uninfected references (Table 2). In-hospital mortality rates for patients with MDR *Acinetobacter* infections (26%) were higher than for susceptible references (18%) and uninfected references (11%). However, only the difference between MDR *Acinetobacter*–infected patients and uninfected patients was statistically significant (Table 2). When controlling for severity of illness with the APACHE score and for underlying disease with the Charlson index in a conditional logistic regression model, association with a longer hospital and ICU length of stay was approximately twice as likely for patients with MDR *Acinetobacter* infection as for susceptible references (Table 3). Multivariable analysis controlling for severity of illness with the APACHE score and underlying diseases with the Charlson index showed a trend toward more deaths associated with infection with MDR *Acinetobacter* than with infection with susceptible *Acinetobacter*, but the difference was not statistically significant (relative risk 2.6, 95% CI 0.3–26.1) (Table 3).

**Table 2:** Matched univariate analysis comparing outcomes of patients with multidrug-resistant (MDR) *Acinetobacter* infection with those with susceptible *Acinetobacter* infection and those without *Acinetobacter* infection, Baltimore hospitals, 2003–2004

Outcome evaluated	MDR *Acinetobacter,* n = 96	Susceptible *Acinetobacter,* n = 91	p values for *MDR Acinetobacter* vs. susceptible, n = 182	Uninfected, n = 89	p values for *MDR Acinetobacter* vs. uninfected, n = 178
Mean length of stay after index day, d	27.5	19.8	0.02	18.6	<0.01
Mean intensive care unit length of stay after index day, d	13.3	6.7	0.04	7.3	<0.01
Mortality rate (%)	26.0	17.6	0.21	11.2	<0.01

**Table 3:** Multivariable analysis of outcomes of patients with and without multidrug-resistant (MDR) *Acinetobacter* infections, Baltimore hospitals, 2003–2004*

Outcome evaluated	MDR *Acinetobacter* vs. susceptible† OR (95% CI)	MDR *Acinetobacter* vs. uninfected† OR (95% CI)
Length of stay, d	2.5 (1.2–5.2)	2.5 (1.2–5.4)
Intensive care unit length of stay, d	2.1 (1.0–4.3)	4.2 (1.5–11.6)
Mortality rate (%)	2.6 (0.3–26.1)	6.6 (0.4–108.3)

*OR, odds ratio; CI, confidence interval. †Models include modified Acute Physiology and Chronic Health Evaluation III score to control for severity of illness and Charlson index to control for underlying disease.

Discordant antimicrobial drug therapy was more common for MDR *Acinetobacter*–infected patients than for susceptible references (91% vs. 65%, p<0.001). When we controlled for severity of illness and underlying diseases, we found that initial discordant antimicrobial drug therapy was not associated with increased mortality rates or hospital length of stay among patients infected with MDR *Acinetobacter* (Table 4). However, patients infected with MDR *Acinetobacter* who were treated with initial discordant antimicrobial drug therapy were >5× as likely to be associated with an increased ICU length of stay.

**Table 4:** Multivariable analysis of discordant versus concordant empiric antimicrobial drug therapy in patients with multidrug-resistant *Acinetobacter* infections, Baltimore hospitals, 2003–2004*

Outcome evaluated	Discordant vs. concordant empiric antimicrobial drug therapy†	
	OR (95% CI)	p value
Length of stay, d	1.6 (0.4–6.5)	0.54
Intensive care unit length of stay, d	5.8 (1.2–27.1)	0.03
Mortality rate (%)	0.7 (0.1–4.5)	0.74

*OR, odds ratio; CI, confidence interval. †Model includes modified Acute Physiology and Chronic Health Evaluation III score to control for severity of illness and Charlson index to control for underlying disease.

### MDR *Acinetobacter*–infected Patients Compared with Uninfected References

Uninfected reference patients were identified for 89 of 96 patients with MDR *Acinetobacter* infections. Reference patients were not identified for 7 MDR *Acinetobacter* patients because there were not enough uninfected patients with extensive hospital length of stay. MDR *Acinetobacter*–infected patients and uninfected references were similar in age and sex. However, patients with MDR *Acinetobacter* infection had higher baseline mean APACHE and Charlson index scores than references (Table 1). Matched univariate analysis of patient outcomes showed that patients with MDR *Acinetobacter* had higher in-hospital mortality rates (26% vs. 11%, p<0.01) and mean hospital and ICU lengths of stay after the index day than uninfected references (Table 2). When we controlled for severity of illness and underlying conditions, we found that MDR *Acinetobacter*–infected patients were more likely to have both longer hospital and ICU lengths of stay than uninfected references (Table 3).

## Discussion

*Acinetobacter* is emerging as an important pathogen in traditional and nontraditional healthcare settings. Its ability to infected healthy hosts and its propensity to develop antimicrobial drug resistance have caused concern among the infectious diseases community. Our study assessed the clinical outcomes of patients infected with MDR *Acinetobacter* compared with outcomes of patients infected with susceptible *Acinetobacter* strains and patients without *Acinetobacter* infections among a large cohort. We demonstrated that patients infected with MDR strains of *Acinetobacter* have longer lengths of stay in both the hospital and ICU than patients infected with drug-susceptible *Acinetobacter* and patients without *Acinetobacter* infection when we controlled for severity of illness. We found a trend toward increased mortality rates among patients with MDR *Acinetobacter* infection. However, the difference was not statistically significant when we controlled for severity of illness.

According to NNIS, *Acinetobacter* species caused 7% of ICU healthcare-associated pneumonias in 2003 compared with 4% in 1986 (p<0.001) (*4*). The proportion of ICU healthcare-associated urinary tract infections and surgical site infections caused by *Acinetobacter* also increased significantly from 1986 to 2003 (p<0.001) (*4*). Furthermore, the proportions of *Acinetobacter* isolates reported to NNIS that were resistant to ceftazidime, amikacin, and imipenem all increased significantly during that period (p<0.001). Healthcare-associated outbreaks of MDR *Acinetobacter* infection have been reported in Asia, Europe, North America, and among US service members injured in the Middle East (*7*–*9*). These findings have brought control of MDR *Acinetobacter* infections to the forefront of discussion.

Investigating the effect of multidrug resistance on clinical outcomes presents multiple methodologic challenges that have been explicitly addressed in our study design. Confounding risk factors associated with mortality rates and antimicrobial drug resistance, such as age, severity of illness, and underlying disease (*18*–*21*) must be controlled for in the study design or analysis. Our results differ from those of researchers who examined outcomes of *Acinetobacter* infections without controlling for these confounders (*14*,*22*,*23*), which makes their findings difficult to interpret. We assessed and controlled for severity of illness and underlying disease with 2 measurements: the APACHE score, which included age, physiologic parameters, and selected underlying diseases; and a separate Charlson index, which included a broader range of underlying diseases. Both measurements have been validated, although the APACHE score has only been studied in its original form (*19*,*21*,*24*). Because patients infected with *Acinetobacter* have worse clinical outcomes than those who are colonized with the organism (*11*), we separated *Acinetobacter* infection from colonization on the basis of standardized, validated Centers for Disease Control and Prevention (Atlanta, GA, USA) NNIS definitions for nosocomial infection (*16*,*25*) and applied them uniformly to MDR *Acinetobacter* and susceptible references. We compared outcomes of MDR *Acinetobacter* infections with those of 2 reference groups and showed an association of MDR *Acinetobacter* infection with both increased hospital and ICU lengths of stay, regardless of the reference group selected. As one would predict on the basis of results of a study by Kaye et al., the effect of multidrug resistance was greater compared with uninfected than susceptible references (*15*).

Because of the lack of a standard definition for multidrug resistance in the literature, we defined multidrug resistance as resistance to all or all but 1 antimicrobial drug class commonly prescribed for treatment of patients with gram-negative infections, with the exclusion of polymyxins (*26*). This definition has 2 advantages. First, it is a strict standard and is readily accepted by clinicians as representative of multidrug resistance. Second, it allows for a clear distinction between susceptible and MDR *Acinetobacter* strains because we excluded isolates that showed intermediate resistance (strains resistant to all but 2 commonly prescribed antimicrobial drug classes).

The association of MDR *Acinetobacter* infections with worse clinical outcomes could be related to discordant empiric antimicrobial drug therapy. Previous studies that examined the effects of delayed concordant antimicrobial therapy on patient outcomes have shown conflicting results (*27*–*29*). We examined this issue and found that patients with MDR *Acinetobacter* infections who received discordant empiric antimicrobial drug therapy were not more likely to die or have a longer hospital length of stay than patients who received concordant empiric drug therapy; however, they were more likely to have a longer ICU length of stay. On the basis of these results, to what extent discordant empiric antimicrobial drug therapy affects clinical outcomes of MDR *Acinetobacter* infection is not clear.

Determining optimal infection control approaches to MDR *Acinetobacter* has been complicated by the lack of agreement on the clinical significance of *Acinetobacter* infections. The Hospital Infection Control Practices Advisory Committee guideline for isolation precautions in hospitals recommends targeting increased infection control efforts toward “resistant bacteria judged by the infection control program to be of special clinical and epidemiologic significance” (*30*). We found that MDR *Acinetobacter* infections are independently associated with increased hospital and ICU lengths of stay. This finding, combined with increased risk for in-hospital transmission of the organism (*31*), supports recommendations to implement aggressive control measures to limit the transmission of MDR *Acinetobacter* in healthcare settings.

Several limitations of this study merit discussion. Because of the lack of available data to calculate a standard APACHE III score for non-ICU patients, we modified the APACHE III score by excluding variables that were unavailable for non-ICU patients. However, our findings support the validity of this scoring system as a measure for severity of underlying illness. Mean APACHE scores were higher in MDR *Acinetobacter*–infected patients than in both reference groups and progressed stepwise from no infection to MDR infection. These findings are expected because drug-resistant infections reportedly occur in sicker patients (*24*,*32*,*33*). Univariate analysis showed that a modified APACHE score was also associated with mortality rates (p<0.001), which further supports its validity as a measure of illness severity.

The lack of available reference patients with similar exposure times to several of the case-patients (5 susceptible references and 7 uninfected references) was a second limitation because we were obligated to exclude unmatched MDR *Acinetobacter*–infected patients from our analysis. These excluded patients typically had prolonged exposure times and tended to have long hospital and ICU lengths of stay after infection. Exclusion of these patients decreased the power of our study and likely biased our results toward showing no difference in hospital or ICU length of stay between the groups. Finally, the lack of a difference in mortality rates, according to multivariable analysis, could mean that MDR *Acinetobacter* are not more virulent than nonresistant strains or that the sample size in this study lacked the power to show a difference.

Our study indicates that infections with MDR *Acinetobacter* are independently associated with the adverse clinical outcomes of prolonged hospital and ICU lengths of stay compared with the outcomes for uninfected patients and those infected with drug-susceptible *Acinetobacter*. This is the first study evaluating length of stay and mortality rates associated with MDR *Acinetobacter* infection while controlling for important confounders such as severity of illness and underlying disease. These data emphasize the need for aggressive infection control strategies to prevent MDR *Acinetobacter* infection and its adverse effects on hospitalized patients.
